# Puzzle game-based learning: a new approach to promote learning of principles of coronary artery bypass graft surgery

**DOI:** 10.1186/s12909-023-04156-w

**Published:** 2023-04-13

**Authors:** Reza Khorammakan, Athar Omid, Mohsen Mirmohammadsadeghi, Ahmad Ghadami

**Affiliations:** 1 Student research committee, School of nursing and midwifery, Isfahan university of medical sciences, Isfahan, Iran; 2 Associate professor, Medical Education Research Center, Department of Medical Education, Isfahan University of Medical Sciences, Isfahan, Iran; 3 Associate professor of cardiac surgery, Isfahan university of medical sciences, Isfahan, Iran; 4 Department of operating room, Nursing and midwifery research center, School of nursing and midwifery, Isfahan university of medical sciences, Isfahan, Iran

**Keywords:** Surgical technologist, Knowledge, Cognitive function, Coronary bypass surgery, Game-based training, Gamification, Puzzle game, Problem-solving, Reasoning

## Abstract

**Introduction:**

Since learning with high educational quality requires an advanced intervention. This study seeks to answer how many puzzles game-based training can improve knowledge and cognitive function of surgical technology students in CABG surgery and its sequence, as well as the tools and equipment used in each stage of surgery and the sequence of their preparation.

**Materials and methods:**

This study was carried out as a quasi-experimental single-group pre-test-post-test, during which, after designing a puzzle game including various stages of surgery (from the preparation of the patient for surgical sutures and the necessary equipment to perform each stage), 18 people from third-year surgical technology students who met the inclusion criteria were entered in the study by convenience sampling method and based on the sample size determined using a similar study and they participated in the test of knowledge and cognitive function, that the validity and reliability were measured, before the intervention and 14 days after the intervention (using a puzzle game). Data were analyzed using descriptive and Wilcoxon statistical tests.

**Results:**

After the withdrawal of 2 people, 15 person (93.80 per cent) of the students were female, the average age of students was 21.87 ± 0.71 years, and 50% (8 people) of them were 22 years old. Also, the average score of the end-of-semester exam of the heart surgery technology course was 15.19 ± 2.30 (the lowest score was 11.25, and the highest score was 18.63), and the score of 43.80% (7 people) of them were in the range of 15.01–17.70, and their average of grade point average was 17.31 ± 1.10 (the lowest grade point average is 15 and the highest grade point average is 19.36) and grade point average 75% (11 people) of students were 16–18. The average scores of knowledge(5.75 ± 1.65 vs. 2.68 ± 0.79) and cognitive performance(6.31 ± 2.57 vs. 2.00 ± 1.09) of students in the post-intervention phase were significantly higher than the pre-intervention phase (P < 0.0001).

**Conclusion:**

The results of the present study showed that the use of puzzle games in CABG surgery training led to a significant improvement in the knowledge and cognitive performance of surgical technology students regarding the stages of CABG surgery and its sequence, as well as the tools and equipment used in each stage of surgery and the sequence of their preparation.

**Supplementary Information:**

The online version contains supplementary material available at 10.1186/s12909-023-04156-w.

## Introduction

Performing surgery requires a combination of cognitive and clinical *skills.* Cognitive skills can be thinking and problem-solving processes and require clinical skills [1, 2]. Clinical training has a decisive role in the process of acquiring cognitive, psycho-motor, and emotional skills [3, 4].

One of the areas of clinical education is the operating room, which is the primary environment for acquiring skills such as patient preparation and drape, setting up the environment. Surgical sterility and the arrangement of the surgical table with the use of surgical tools in the order of the procedures and their use are for surgical technology students [5]. In this environment, conducting training in surgeries such as CABG surgery faces limitations due to its sensitivity and complexity, patient safety concerns, insufficient training time, stressful environment, and special regulations in the cardiac operating room [6–9]; Therefore, quality cognitive training outside the operating room is critical so that students can be sufficiently prepared to acquire clinical skills in this environment.

Knowledge, the lowest level of Bloom’s cognitive skill, refers to maintaining specific and separate pieces of information, such as the sequence of performing skills [10]. Following the research of the last few years to justify the cognitive performance of students who had problems showing their capabilities and were considered weak students, the importance of cognitive performance in academic performance was determined [11].

Education is necessary for the development of people’s cognitive performance. In the effective learning process, learners’ understanding and cognitive abilities such as fast thinking (processing speed), keeping the information in mind (working memory), flexible response to work goals (cognitive control) and dealing with new problems (argument) were promoted [12–15].

Lo-Cariaga et al.‘s study (1996) showed that non-cognitive characteristics were strengthened when they experienced problem-based learning [16]. In the traditional method of education, there has been less interaction between the learner and the educational content, which has led to the loss of the learner’s interest in learning concepts [17]. Since traditional training methods have not been able to provide adequate training for surgical technology students [18], and due to the advancement of technology and educational technology, high-quality learning requires an advanced training program and intervention [19]. Since humans can learn and understand 50% of what they hear and see, and 30% of what they see, learners need tools to visualize training content for effective learning [19]. When training is visual, the spatial awareness of learners is increased and leads to a better and deeper understanding of concepts, increasing reasoning skills and cognitive performance; Also, “the learning process is facilitated when it is done in an entertaining method” [20, 21].

Furthermore, since games can involve a wide range of people in individual and social learning activities, learners can learn and practice essential skills and basic knowledge through them [23–31]. In addition to providing a context for learning knowledge and skills, educational games can also play the role of a coach and professor [32]. Piaget and Vygotsky each considered playing important for learning and development. However, theories differ somewhat on specific details. For example, Piaget describes play as the primary motivation for pleasure, but Vygotsky points to a broader range of motivations for engaging in play. Also, according to Piaget, play is an opportunity to reflect and reinforce what has already been learned, while according to Vygotsky, play is a vital tool for learning new concepts. Piaget’s and Vygotsky’s ideas about play, although somewhat different, point to the same great potential of games as tools to support learning and development [31]; Therefore, digital games were proposed as a valuable tool for learning, increasing cognitive and perceptual skills and motivating students [20, 21, 33–40, 80].

Puzzle game-based learning has many applications in the education and learning of computer [41], chemistry [42], anatomy [43], pharmacy [44], physiology [20, 45], pathology [21, 36], neurosurgery [46] and mathematics [47]. The Institute of Decision-Making Sciences considers puzzle-based learning as an introduction to critical thinking and problem-solving [48]. According to Michalewicz, puzzles should meet at least one of the criteria of generality(providing the player with the ability to solve real problems in the future), simplicity (the puzzle and its solution are quickly recorded in the player’s memory), entertainment (encouraging the player’s interest in solving the puzzle) and Eureka (each piece must be placed in its place, and after placement, the player must be given immediate feedback and reward) [49].

The benefits of puzzle game-based education include turning passive learners into active ones, improving interest, satisfaction, and academic performance, improving critical thinking and problem-solving skills in other real-life areas instead of covering content and encouraging analogical and clinical reasoning. Comprehensive stimulation to the practical application of learned knowledge with their combination. Increasing concentration and stimulation of comprehensive memory to consolidate and better understand concepts. Highlighting the main concepts, acquiring cognitive skills, and improving executive performance(due to the need to formulate strategies, rearrange and planning), pointed out the transformation of the education process from instructor-oriented to learner-oriented [17, 19, 21, 36, 37, 49–52, 48, 81].

Cardozo et al. (2021) found that, according to students, the online digital version of the heart cycle puzzle is helpful for their learning [21]. Coelho et al. (2020) knew that the craniosynostosis surgery puzzle might be a complementary tool for education because it allows the possibility of observing complex surgeries with a three-dimensional, dynamic view and in a realistic environment without causing any risk to the patient gives to students [46, 54, 56]. Nouchi et al.'s study (2013) showed that the Tetris puzzle game improves executive functions, working memory, and processing speed in healthy young people compared to the brain training game [53].

If the duties of a surgical technologist in a specific surgery be taught to the student in a step-by-step, visual way and in an environment that promotes problem-solving skills and executive function, he/she will be able to mentally visualize of the surgical steps in the clinical environment and retrieval of classified image information, have shown good performance and will become an efficient force for the operating room in the future; therefore, this study seeks to answer how many puzzles game-based training can improve knowledge and cognitive function of surgical technology students in CABG surgery and its sequence, as well as the tools and equipment used in each stage of surgery and the sequence of their preparation.

## Materials and methods

### Study design

This study was conducted as a semi-experimental single-group pre-test-post-test during three phases of the puzzle game scenario design, puzzle game construction, and determining the effectiveness of the puzzle game on the level of knowledge and cognitive performance.

### Ethical considerations

First, the code of ethics was obtained from the regional ethics committee in medical science research. Then, the process of conducting the study and its objectives were explained by the researcher through WhatApp messenger to each of the third-year undergraduate students of surgical technology who met the criteria for entering the study—completed the online informed consent form (in WORD file format) for each of them. It was completed and delivered to the researcher through App messenger as a WORD file.

### Sample size

Considering that to evaluate the effectiveness of this intervention, at least 15 volunteer surgical technology students would be needed(calculated using formula 1), and based on result of similar study[54] and estimated losses(20%), a sample of this size would allow us to detect a somewhat large effect size, on the order of 2.07(d), with a confidence interval of 95%(z_1_) and power of 80%(z_2_), with a standard deviation of 2.27 score for pre-intervention phase(s_1_) and standard deviation of 1.74 score for post-intervention, this sample size would allow us to find mean differences of 4.72 scores.

$${n} = \frac{{\left(Z1+Z2\right)}^{2}({S1}^{2}+ {S2}^{2})}{{d}^{2}}=15$$

**Formula 1: calculation of sample size**

## Study phases

### Puzzle game scenario design

At first, the research team (consisting of a cardiac surgeon with 20 years of experience in coronary artery bypass graft surgery (CABG), an assistant professor in the surgical technology department, an assistant professor in the medical education department, a master’s student in surgical technology with a history of presence and clinical activity in coronary artery bypass Graft surgery) studied the educational curriculum and authoritative books [5, 55–57] to investigate the educational needs of surgical technology students and then a 10-member panel of experts consisting of professors from the department of surgical technology of the university of medical sciences (with at least 5 years of experience teaching theoretical and clinical courses in CABG surgical technology, at least a bachelor’s degree in surgical technology), surgical technologists working in specialized cardiac hospitals with at least 10 years of activity in CABG surgery as a scrub surgical technologist, cardiac surgeons with at least 20 years of experience in CABG surgery, professors of the medical education department of the university of medical sciences and surgical technology students (with a history of presence and clinical activity in coronary artery bypass graft surgery), was formed and during the holding of 2 sessions, the theoretical and clinical educational needs of surgical technology students in the topic of CABG surgery, the problems in the effective education of students and the results of the educational needs assessment conducted by the research team were discussed and based on the results of the expert panel meetings and also using reliable surgical technology books [5, 56–58], the research team designed the coronary bypass graft surgery puzzle game scenario.

In the scenario, different stages of CABG surgery use the on-pump method, which includes primary preparation, secondary preparation, patient drape, and preparation of sterile cardiopulmonary pump tubes. The harvest of the great saphenous vein, the harvest of the left intrathoracic artery (LIMA), sternotomy, Pere cardiotomy, arterial and venous cannulation, distal and proximal anastomosis and intrathoracic artery to the left anterior descending coronary artery (LAD), patient separation from the cardiopulmonary pump and sternotomy closure, and the tools and supplies needed to perform each stage of surgery, was included.

In the next stage, the game scenario was provided to the experts panel for content validity and their opinions were obtained regarding the compatibility of the designed scenario with the procedures of surgery in the operating room of Isfahan, Iran, and the content validity of the scenario was approved by all the members of the experts panel (10 person) arrived. The scenario was written in accordance with the sequence of CABG surgery steps and the name of the surgical tools used was changed according to their standard names, including the opinions of the experts panel members regarding the CABG surgery puzzle game scenario.

### Making a puzzle game

At first, an expert team with 3 years of experience in designing, programming and building high-quality online and offline educational games was added to the research team to create a CABG surgery puzzle game according to the scenario designed in phase 1–4.

This team designed the initial and trial version of the game within a period of 3 months and the trial version was given to the experts panel who were also present in phase 1–4 to check the content validity, and the panel members reviewed all the parts of the game in 3 sessions. Among the comments of the expert panel members, it is possible to mention improving the quality of the puzzle images, adding the heartbeat avatar, adding images to some puzzle pieces, and changing the background screen of the game, which have been applied in the game, and the edited version of the game is again available to the expert panel members and the puzzle game was approved by all panel members.

**Fig. 1:**
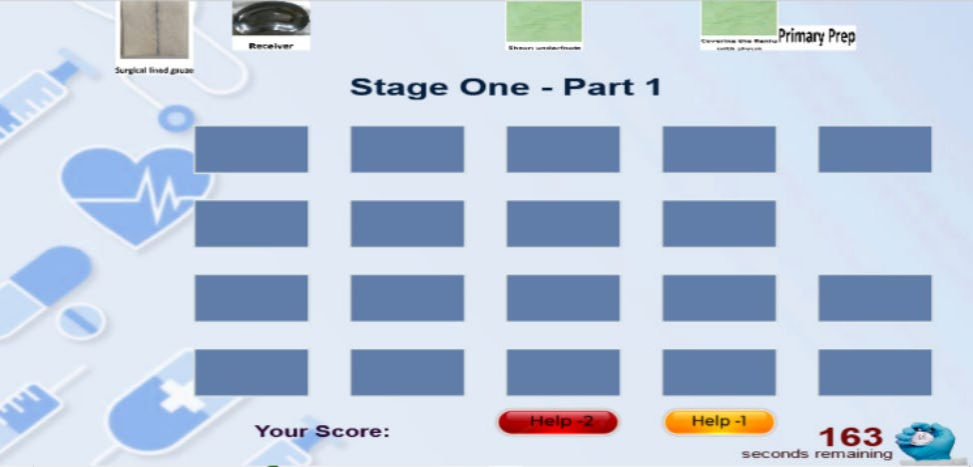
CABG surgery game page

The designed online puzzle game includes 3 stages and 12 sections with a heartbeat avatar representing the duties and functions of a scrub surgical technologist in coronary artery bypass surgery (CABG). How to play the Coronary Artery Bypass Surgery (CABG) puzzle game: The player enters the CABG surgery game space (Fig. 1) and, in total, during three stages, with 183 image pieces arranged in order with the stages of CABG surgery and the supplies needed for each stage. He will face surgery, and he must put the pictures and pieces together and reach the end of the game within 28 min. The first stage of the game consists of 5 parts: primary prep (9 pieces), secondary prep (10 pieces), drape (9 pieces), arterial-venous line (4 pieces), and harvest of the great saphenous vein (16 pieces). In the first stage, it is 7 min. The second stage of the game consists of 11 parts: Sternotomy (9 pieces), pericardiotomy (3 pieces), harvesting of the left intrathoracic artery (LIMA) (14 pieces), cutting the lines (3 pieces), releasing the LIMA (5 pieces), and pericardial traction. (5 pieces), arterial cannulation (11 pieces), venous annulation (7 pieces), placement of other cannulas (10 pieces), distal anastomosis (15 pieces), and LIMA to LAD anastomosis (8 pieces).

**Fig. 2:**
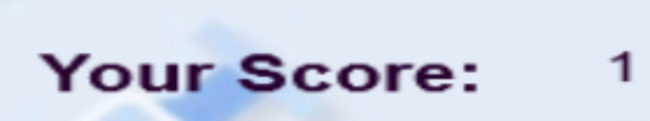
A positive point for the player

**Fig. 3:**
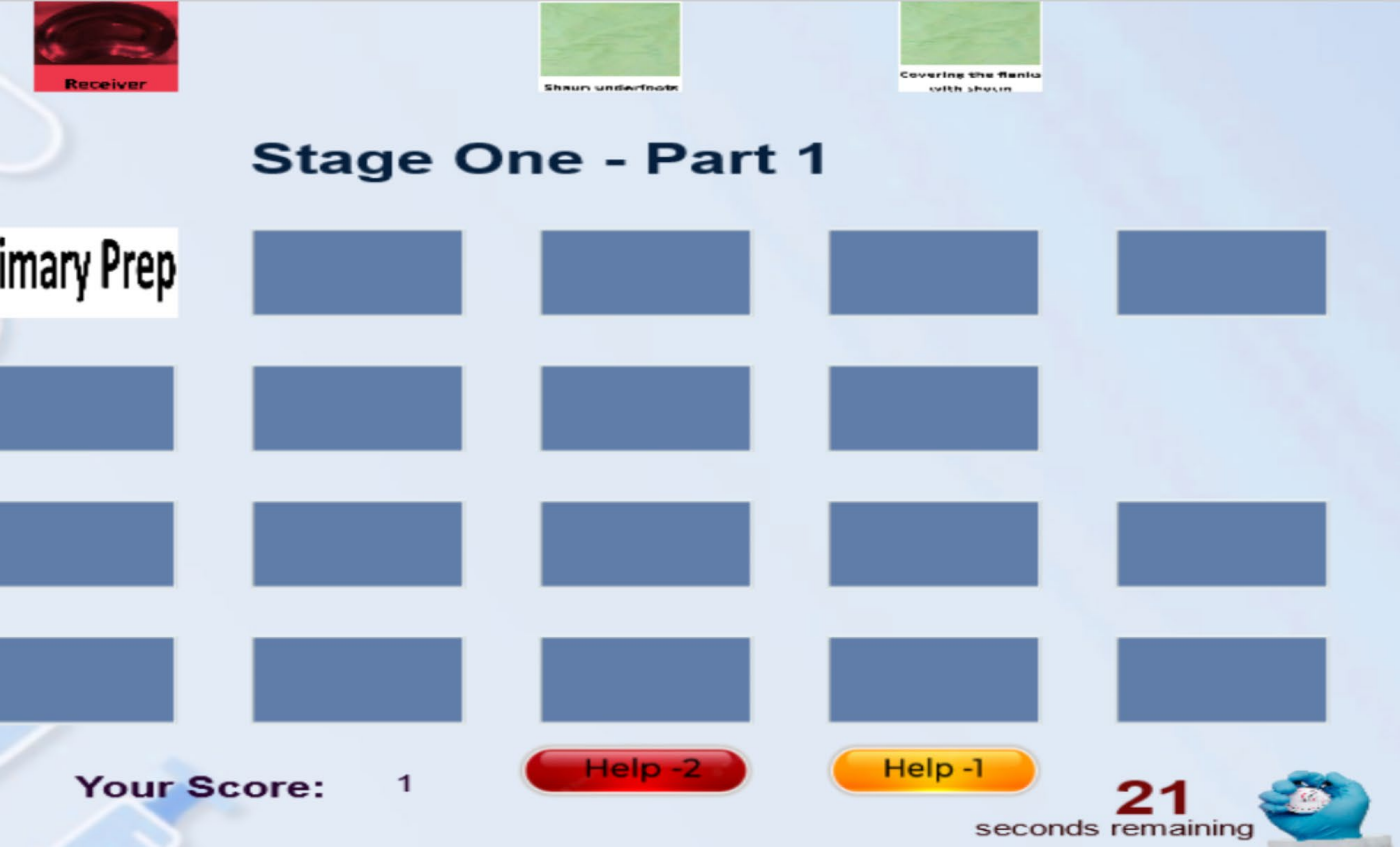
The player’s first wrong choice

**Fig. 4:**
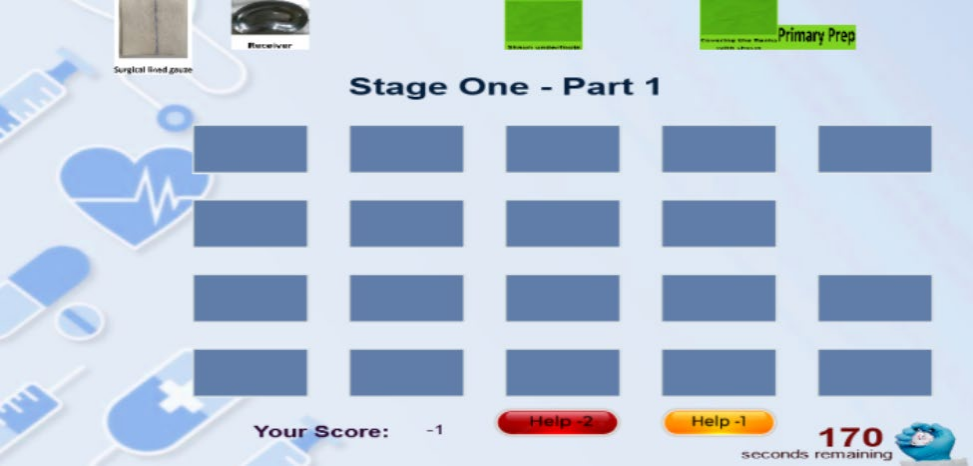
The player’s second wrong choice

The first is 14 min. The third stage of the game consists of 4 parts of proximal anastomosis (16 pieces), removal of cannula (6 pieces), examination of the sternum bed and insertion of the chest tube and pacemaker electrode (11 pieces), and sternotomy sutures (12 pieces). The game in the first stage is 7 min. The player must first click on one of the right pieces and then click on the right place so that the piece is placed in its place, and for every piece that he puts correctly in its proper place, he gets a positive point (Fig. 2).) and he can choose an image and piece wrongly or put it in the wrong and inappropriate place only once, and no points will be deducted from him (Fig. 3), but if he makes a mistake more than once, for every wrong choice again, One point will be deducted from him (Fig. 4).

**Fig. 5:**
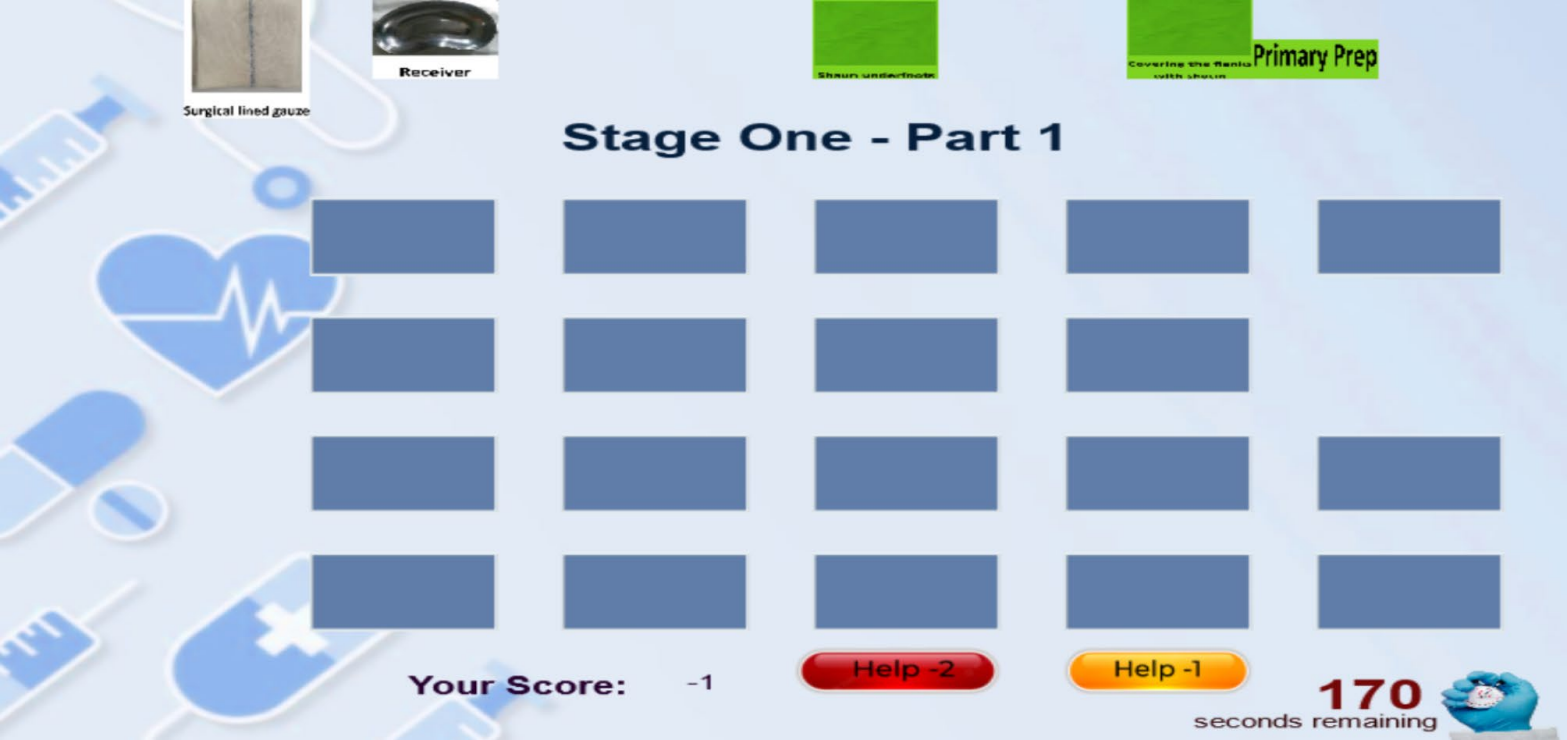
Using the first type of guidance for the first time

**Fig. 6:**
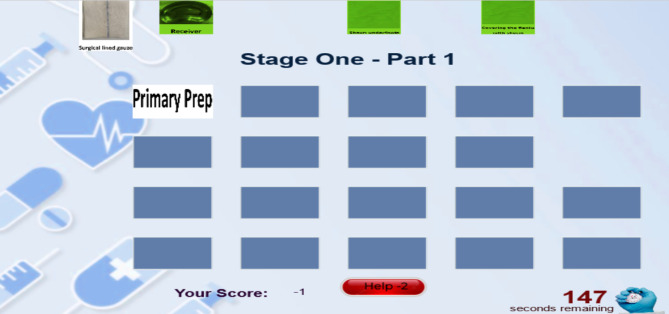
Using the first type of guidance for the second time

**Fig. 7:**
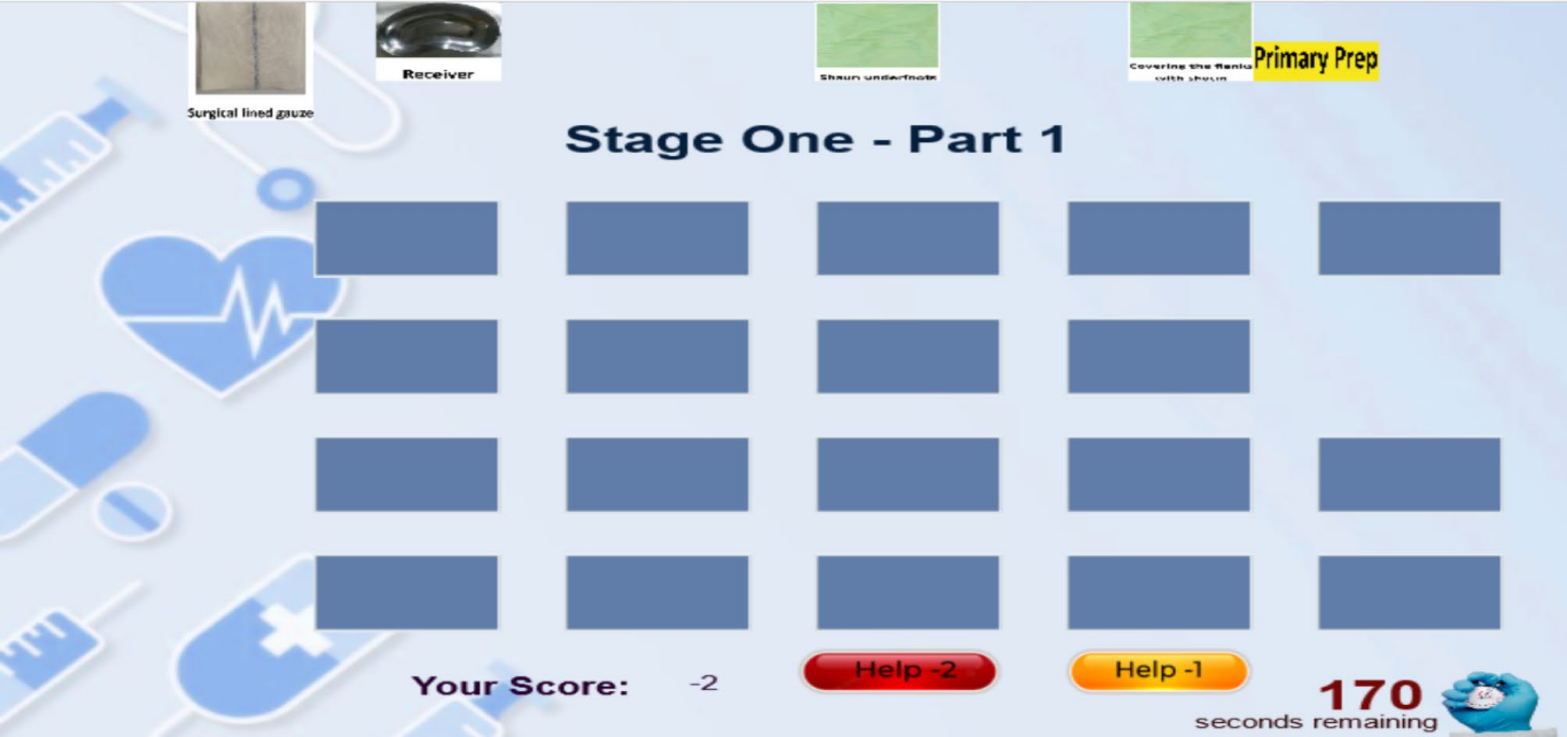
Using the second type of guidance for the first time

**Fig. 8:**
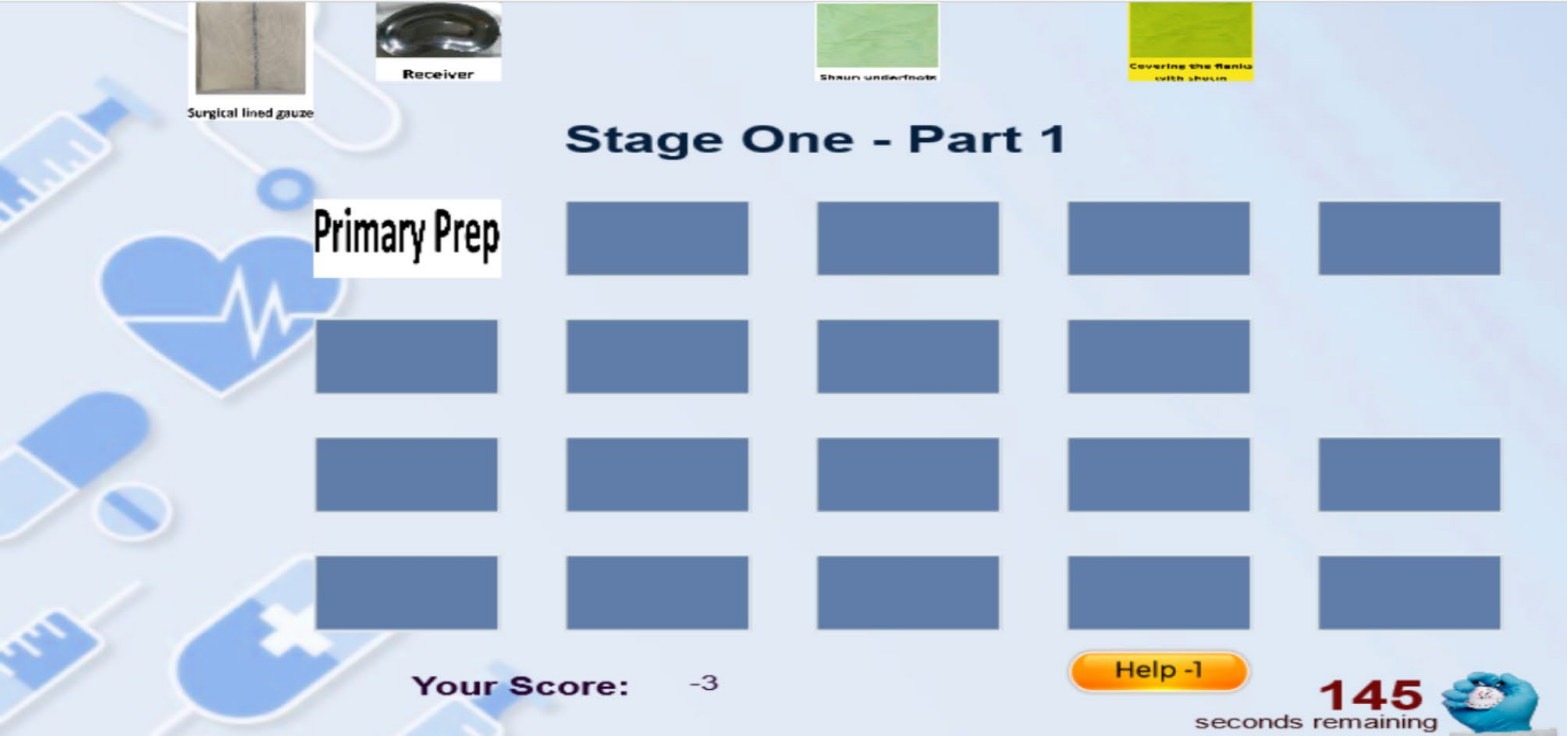
Using the second type of guidance for the second time

**Fig. 9:**
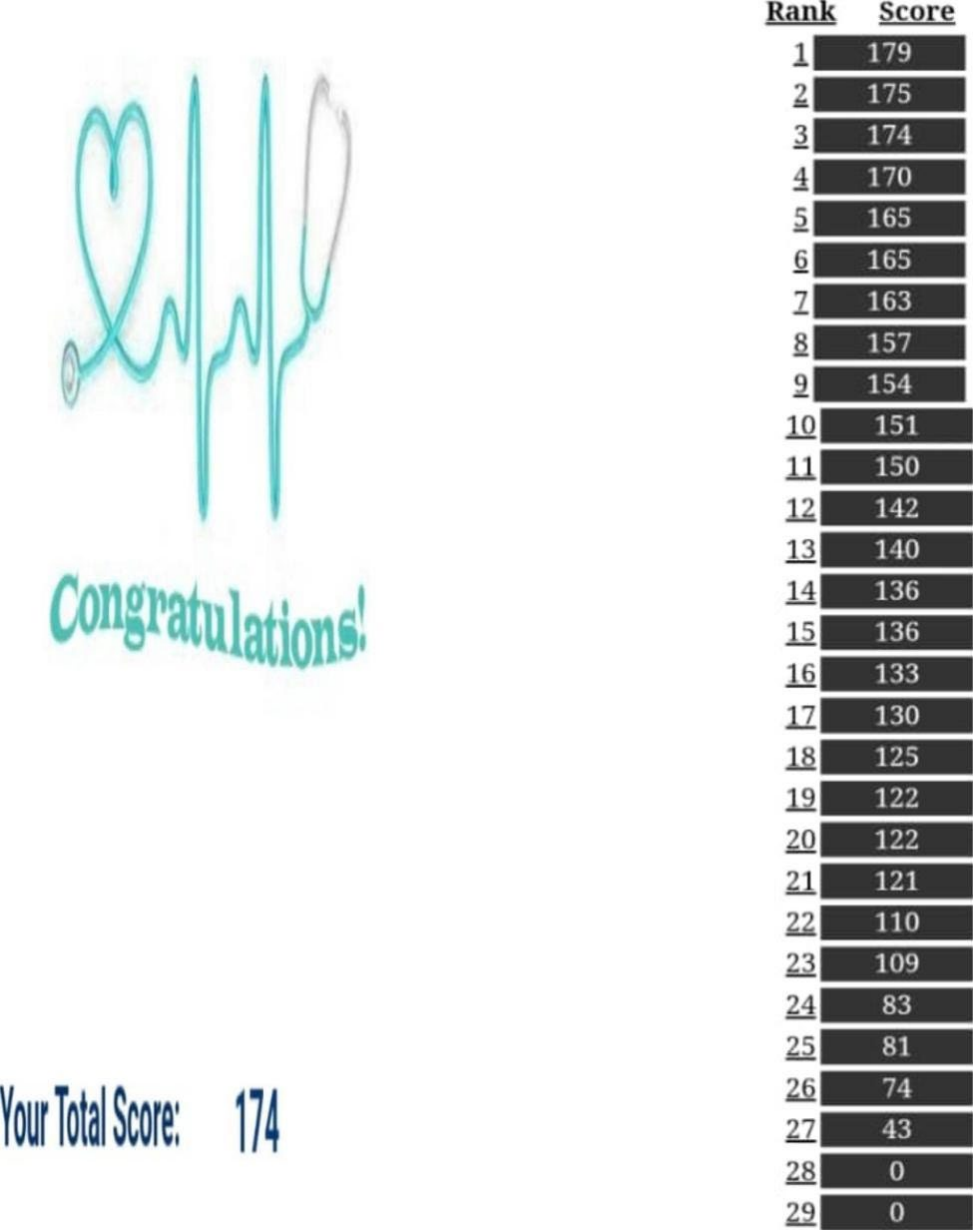
Score and rank of the player at the end of the game

Since the surgery includes a series of connected and consecutive actions, selecting parts is not the user’s responsibility. (for example, the user cannot first place the part corresponding to position number 6 and then the part corresponding to position number 3) could we put it in its proper place? The empty places should be filled in order with appropriate pictures.). The user can ask for guidance and help if needed, and in this part of the system, he is offered two types of guidance (the first and second type guidance). The user can choose any of them at each game’s stage, for 1 and 2 points will be deducted from the user according to the selection order and the type of guidance given. In the first type of guidance for the user, three pieces are colored green, of which the correct piece is one of them. The user can choose the correct piece from among them, and in the second type of guidance, the correct piece is colored yellow. The player places it in the position (Figs. 5, 6, 7 and 8). At the end of the game, the user’s score and rank among other players will be determined (Fig. 9).

### Determining the effectiveness of the puzzle game on the level of knowledge and cognitive performance

At this stage, 18 students in the third year of surgical technology, who meet the criteria for entering the study, were selected by an easy (available) Convenience sampling method. After a full explanation of the process of conducting the study and its objectives by the researcher through WhatApp messenger and completed the online informed consent form (in WORD file format) of the study was started.

The inclusion criteria included careful reading and signing of the informed consent form to participate. In the study, they had a computer, tablet, or mobile phone with the ability to connect to the Internet to play the CABG surgery puzzle game and have the Internet with a suitable speed to play the CABG surgery a puzzle game and study theoretical topics. The instructor expressed them of CABG surgery technology, absence in the CABG surgery technology theory classroom, lack of experience as a surgical technologist in CABG surgery, lack of viewing videos and images of CABG surgery procedures, currently studying in the third year of a continuous undergraduate degree in surgical technology and standards. Exclusion criteria included unwillingness to continue participating in the study for any reason and at any stage of the research, failure to complete the puzzle game at least two times, and game over in the CABG surgery puzzle game more than three times.

In coordination with the student’s representative, a date was set to perform online tests of knowledge and cognitive function before the intervention (pre-test). The security of the test by the maintain, the duration of the test was reduced found that there were no question numbers and options for the examinees. The order of the options was also different for the students. After the pre-test, the researchers introduced the puzzle game and how to do it to all the students through WhatApp messenger, and then the students played the game for 14 days. They had to reach the end of the game successfully at least twice. To ensure students had reached the end of the game, each student had to send the researcher a photo of their score and leaderboard pages. The interval between the dates of the pre-intervention and post-intervention tests were two weeks to prevent recall bias [59–63]. After two weeks of the pre-intervention tests, all the students who had completed the CABG surgery puzzle game at least two times to the end, compared to performing the online tests of knowledge and cognitive function after the intervention (post-test). The security of the online test by the maintenance, the duration of the test was reduced, and there were no question numbers and options for the examinees and the order of the options was also different for students.

## Data collection tools

In this study, a researcher-made test set includes a question section to measure knowledge (including multiple-choice questions) and a question section to measure cognitive performance (including puzzle scenario questions) of students in the role of a scrub surgical technologist in coronary artery bypass surgery. CABG) in the stages of primary prep, secondary prep, patient drape, preparing sterile cardiopulmonary pump tubes, great saphenous vein harvest, intrathoracic artery harvest, chest opening, pericardial opening and traction, arterial and venous cannulation, Distal and proximal anastomosis and thoracic artery to the left anterior descending coronary artery, separation of the patient from the cardiopulmonary pump and star anatomy suture, and preparation of appropriate surgical instruments for each stage and their delivery to the surgeon in the appropriate sequence was used.

The students’ knowledge test consisted of 13 four-choice questions and the students’ cognitive performance test consisted of 17 puzzle scenario questions. The correct answer to each question had a positive score and a wrong answer or failure to answer each question had a zero score; therefore, the minimum score of the student from this test was zero, and the maximum was 13 and 17 points for knowledge and cognitive performance, respectively. The content validity of the knowledge test questions by the measure (13 four-choice questions) and cognitive performance (17 puzzle scenario questions) was given to 5 faculty members of the medical sciences universities in the country. The face validity and content of the questions were evaluated using the index Louche (CVR), and Waltz and Basel (CVI) were investigated. Validity results indicated that the value of CVR and CVI for 13 questions in the field of knowledge and 17 questions in the field of cognitive performance were 0.99 and 0.79, respectively, and the content validity of the the instrument used in this study was confirmed.

Milkman’s checklist and Blueprint were used to measure the face validity of the questions of knowledge and cognitive function tests. The face validity results indicated that the questions of both tests have appropriate face validity.

A preliminary study was conducted to measure the reliability of the test questions using Cranach’s alpha method. During that, test questions were given to 14 students in the fourth year of the bachelor’s degree and the first year of the master’s degree in surgical technology.

Cornbrash’s alpha coefficient was calculated using the 16 SPSS software. The obtained results showed that the Cornbrash’s alpha coefficient for the questions in the field of knowledge (13 questions), the field of cognitive performance (17 questions), and the real test (30 questions) was equal to 0.787, 0.979, and 0.963, respectively, and showed the reliability of the questions.

## Statistical analysis

The data were analyzed using descriptive statistical tests (prevalence and frequency percentage, mean and standard deviation), Shapiro and Wilcoxon, and SPSS software version 16.

## Results

After reviewing the data from 18 students, the data of 2 students were removed due to playing the puzzle game once and incomplete completion of the demographic questionnaire. The data of 16 students were analyzed. The results of demographic variables (Table 1) showed that 15 people (93.80 per cent) of the students were female, their average age was 21.87 ± 0.71 years, and 50% (8 people) of them were 22 years old.

**Table 1:** Distribution of demographic variables

Demographic information	Frequency (percent frequency)	mean ± Standard deviation
Gender Female Male Total	15(93.80) 1(6.20) 16(100)	
**Age** **21** **22** **23** **Total**	5(31.30) 8(50) 3(18.70) 16(100)	21.87 ± 0.71
**Heart surgery technology score** **10-12.50** **12.51-15** **15.01–17.50** **17.51-20** **Total**	2(12.50) 5(31.30) 7(43.80) 2(12.50) 16(100)	15.19 ± 2.30
**grade point average** **16–18** **18–20** **Total**	11(75) 5(15) 16(100)	17.31 ± 1.10

Also, the average score of the end-of-semester exam of the heart surgery technology course was 15.19 ± 2.30 (the lowest score was 11.25, and the highest score was 18.63), and the score of 43.80% (7 people) of them were in the range of 15.01–17.70, and their average of grade point average was 17.31 ± 1.10 (the lowest grade point average is 15 and the highest grade point average is 19.36) and grade point average 75% (11 people) of students were 16–18.

First, the normality of the data distribution was checked using the Shapiro test to compare the average scores in the field of knowledge before and after the intervention Wilcoxon test was used for the scores. The results showed that average scores of the knowledge domain before and after the intervention were 2.68 ± 0.79 and 5.75 ± 1.65, respectively, and significantly, the average scores after the intervention were higher than before the intervention. P-value = 0.0001). (Table 2)

**Table 2:** Average knowledge scores

Knowledge	mean ± Standard deviation	Lowest score	Highest score	Shapiro-Wilk	Wilcoxon
**Before intervention**	2.68 ± 0.79	2	4	P < 0.05	P = 0.0001
**After intervention**	5.75 ± 1.65	3	10		

To co-first, the normality of the data distribution was checked using the Shapiro test to compare the average scores of the cognitive function domain before and after the intervention Wilcoxon test was used to compare the scores.

The results showed that the average cognitive function scores before and after the intervention were 2.00 ± 1.09 and 6.31 ± 2.57, respectively, and significantly, the average scores after the intervention were higher. Intervention (P-value = 0.0001). (Table 3)

**Table 3:** Average scores of cognitive function

Cognitive function	Standard deviation ± mean	Lowest score	Highest score	Shapiro-Wilk	Wilcoxon
**Before intervention**	2.00 ± 1.09	1	4	P < 0.05	P = 0.0001
**After intervention**	6.31 ± 2.57	4	15		

## Discussion

This study sought to answer the question of to what extent puzzle game-based training can improve.

the knowledge and cognitive function of surgical technology students in CABG surgery. The results showed that surgical technology students’ knowledge and cognitive function improved significantly after playing the CABG surgery puzzle game. This study was the first one that examined the effectiveness of using the puzzle game-based teaching method in CABG surgery for surgical technology students.

Previous studies have shown that adding elements such as in-game advice and feedback, adding prompts for players to explain or reflect during the game, and creating competition (by showing the player’s score compared to other players) improves learning [64, 65]. In the CABG surgery puzzle game, elements such as a heartbeat avatar provide feedback to the player by providing positive and negative points, guidance, and explanation to achieve the puzzle.

the solution, showing the player’s score at the end game. Moreover, his rank among other players were used.

One of the main reasons for supporting the potential of digital games for learning are there ability to engage in cognitive interaction with learners [66, 82], in which the learner engages in cognitive processes during learning, including selecting relevant information from the game and mentally sorting it into a coherent structure. Moreover, integration is related to previous knowledge and improvement [64]. In the CABG surgery puzzle game, to solve the puzzle, the player must analyze in his mind different pieces of the puzzle. Each of them showed a stage of the CABG surgery and merged it with his previous knowledge about this surgery and chose the puzzle pieces correctly in He placed the right place.

The results of a semi-experimental single-group pre-test-post-test study by Hannani et al. (2019) showed that the mean knowledge scores of surgical technology students in the post-intervention phase (spinal fusion surgery training using games) (17.47) were significant. It is more than the stage before the intervention (10.93) (P = 0.001) [66].

The semi-experimental study of Amir Alavi et al. (2016) The results showed the average scores of knowledge of tracheobronchial anatomy of anesthesiology resident students of Gilan University of Medical Sciences. Before the intervention in the control group (traditional bronchoscopy training), it was equal to 17.82 ± 55.83 in the intervention group (bronchoscopy training). Using simulation web software) it was equal to 66.33 ± 13.71; The scores after the intervention in control and intervention groups were 90.83 ± 10.84 and 119.17 ± 14.43, respectively. There was a significant difference between the average scores of tracheobronchial anatomy knowledge of anaesthesia resident students after the intervention in the intervention group (bronchoscopy training using web-based software similar to compared to the control group (bronchoscopy training by traditional method) (P < 0.0001) [67].

In the semi-experimental study of Akbari et al. (2021), the results showed the average knowledge scores of surgical technology students in the intervention group (teaching surgical table arrangement using games). (6.70 ± 0.72) were significantly higher than the control group (teaching surgical table arrangement using the speech method was (5.90 ± 1.71) (P = 0.040) [68].

In a systematic review by Tavares (2022), the results of a review of 17 articles examined interventions and topics with increased educational complexity that required increased levels of knowledge retention and critical thinking, such as nursing theory and complex clinical skills. Furthermore, topics including game-based learning approaches, student experience and participation, the impact of game-based learning on students’ learning and knowledge retention, and the use of a wide range of learning assessment methods such as quizzes, escape rooms, and serious games, showed that, the game-based teaching method was accepted by the students. They confirmed its widespread use in the nursing curriculum. Most studies reported increased student knowledge and learning when using game-based learning, although time-limited games can often increase student anxiety. In the end, the researchers concluded that game-based learning is an essential alternative to traditional teaching methods; however, the frequent use of game elements and their limited long-term effects may limit their widespread use in nursing education [69].

In a systematic review by Ozdemir et al. (2022), the results of the review of 46 articles showed that game-based learning is used for many different subjects in nursing education. Simulation games are the most widely used type of game, and game-based learning facilitates the achievement of learning outcomes mainly in the cognitive domain (including knowledge, understanding, application, analysis, synthesis, and evaluation). In the end, the researchers concluded that game-based learning is a helpful method to achieve learning results, mainly in the cognitive domain, with some positive and negative aspects. Further research should investigate the effects of games on emotional and behavioural learning outcomes and the use of games to evaluate learning outcomes [70].

The results of Gudadappanavar et al.‘s study (2021) showed that the average post-test scores of second-year MBBS students in the intervention group (teaching using games) (33.17 ± 2.93) were significantly higher than the control group (teaching by lecture method) (2.82). ±28.66) was (P < 0.001) [71].

In the study of McCarroll et al. (2009), the results showed that the average scores of the students in the intervention group (teaching muscle anatomy through lectures and games) that was 5.82% higher than those of the control group (teaching by lectures). However, the difference is that there is no statistical significance (P > 0.05) [72].

Huang et al. (2017) showed that mobile and computer video games improve undergraduate students’ cognitive performance, learning, and memory [73]. The results of Farzad et al.‘s study (2021) showed the average post-test scores of organizational performances (one of the areas of cognitive performance) of preschool children in the intervention group (teaching using Kiko’s Thinking Time game) (56.10) was significantly higher than the control group (teaching Russian to speak) (25.17) (P < 0.001) [74].

Using the puzzle game, as a new educational tool, aims to make creative decisions, create and improve self-confidence in solving problems, stimulate comprehensive memory to consolidate and better understand concepts, highlight the main concepts, and acquire and improve cognitive skills and functions [75].

The results of Padmaja et al.‘s study (2019) also showed that the average comprehension scores of students in the intervention group (teaching concepts of pathology using visual puzzles) (4.46) are significantly higher than the control group (teaching by traditional lecture method) (3.58). P = 0.001) [22], as well as the study of Haripriya et al. (2019), in which the average comprehension scores of students in the intervention group (teaching concepts of pathology using a bingo puzzle game) it was significantly higher than the control group (3.65 ± 0.85 vs. 2.64 ± 0.91) (P < 0.05) [37].

According to Mikhailovich, educational puzzles should include at least one of the four criteria of generality (through the puzzle and solving it, the reader can solve future problems and riddles in real life), simplicity (puzzles and their solutions are easy to remember) fixed and easy to remember), Eureka (the puzzle being exciting and having simple solutions so that solving the puzzle is interesting for the learner) and entertainment (the environment of the puzzle and solving it should be fun and enjoyable) have an impact on applying comprehensive learning [49]. The CABG surgery puzzle game was also designed to have a fun environment and a simple solution for learners to fix the game the solution in their memory and use it in the future to help the CABG surgery team in the hospital environment.

The results of the present study also showed that education based on the puzzle game has significantly led to the improvement of one of Bloom’s cognitive skill areas, that is, the knowledge of surgical technology students in CABG surgery, and with the study’s results by Plant et al. (2019) showed that anatomy education with a 3D puzzle in a virtual reality environment, it can be a valuable complementary tool for traditional anatomy teaching and learning because solving a 3D puzzle in a virtual environment and receiving feedback from solving each part of the puzzle, the student can improve The level of self-knowledge should help [43]; Patrick et al.‘s study (2018) in which the average knowledge scores of pharmacy students who used the word puzzle game for self-learning the basics of pharmacy was 52.69 [44],

Cardozo et al.‘s study (2016) showed that the average knowledge scores of medical students in the intervention group (teaching the heart physiology cycle using puzzles) were higher than those in the control group. (teaching the heart physiology cycle using the lecture method) [45] and Barclay’s study and colleagues (2011) in which the knowledge scores of pharmacy students in the field of cardiac drugs (19.2 vs. 5.1 per cent) (P < 0.001). Infectious diseases (10.3 vs. 5.1 per cent) (P = 0.006) ) in the stage after playing card games, Cardiology Go Fish and Infectious Diseases Gin Rummy was significantly higher than the stage before playing the game [76].

Among the reasons for adaptation, visual education, along with the gasification approach, leads to a better and deeper understanding of learning. Students’ knowledge is improved by discovering the relationships between the components of each phenomenon (surgery, anatomy). The student is stabilized, and their recall is facilitated when applying the material in the natural environment and when taking the test. The concept of the brain’s cognitive function has been the focus of neuroscientists and neuropsychologists. However, recently, researchers have been drawn to the function of the brain in the learning process. Studies have shown that learning takes place during the change in the organization of cognitive functions of the brain, and Quality education leads to better cognitive performance and effective inclusive learning [12–15]. Cognitive performance has different dimensions and scopes based on the level of complexity, including feeling (multi-sensory), perception, movement, and construction skills (copying, drawing), and attention and concentration (selective attention, sustained attention/awareness). Memory (working memory, episodic/declarative memory, procedural memory, semantic memory, and prospective memory), executive function (critical thinking, reasoning, problem-solving), processing speed (mastery, encoding, and tracking), and language/verbal skills (Naming, reading, and understanding [83, 84]. Cognitive performance is the second dependent variable in the present study and the results showed that the use of the CABG surgery puzzle game has significantly improved the cognitive performance of surgical technology students, and with the results of the study by Molyana et al. (2022), which showed that 32 knowledge In the stage before the intervention (playing the puzzle game), the student had low cognitive performance, and eight students had sufficient cognitive performance, and after the intervention, One student had good cognitive performance, 26 students had high cognitive performance, and 13 had very high cognitive performance. Students’ cognitive performance in the stage after the intervention (playing the puzzle game) was significantly higher than in the stage before the intervention. (P < 0.01) [12] a double-blind, a randomized controlled trial study by Nunci et al. (2013) showed that the brain training game improved executive functions, working memory, and processing speed in young people and the Tetris puzzle game led to the improvement of attention and ability. It becomes auditory-spatial. Also, this study provided scientifically evidence that the brain training game has beneficial effects in improving cognitive functions, including executive function, working memory, and processing speed in young people.

The results of the study by Chang et al. (2021) showed that the bingo puzzle game had more effects on increasing the learning motivation of 86 third-year undergraduate students in the field of business, while the so creative mobile application promoted knowledge sharing and critical thinking. In addition, both teaching methods positively affected learning outcomes through common mechanisms, including focused attention, brainstorming, active participation, interaction, and logical thinking. There was a significant interaction between motivation, knowledge sharing, and critical thinking [77]. Kobal et al.‘s (2015) study showed that students who experienced the puzzle game course, they have developed their problem-solving strategies and basic reasoning skills. The average scores of their problem-solving and reasoning skills in the post-intervention stage (using a reasoning puzzle game) were better than the pre-intervention stage [78], Bruker et al.‘s study (2019) in which the average cognitive performance scores of healthy people aged 50–93 in all domains (reasoning, focused and sustained attention, information processing, executive function, working memory, and episodic memory) after playing the number puzzle game is significantly higher than before playing the game (P < 0.0004) [79]. 

Among the reasons for adaptation, visual education, along with the principles of gamification, leads to improving reasoning, perception, cognitive and problem-solving skills. The use of puzzle games for education, as a new visual educational method, can also Encourage learners to think critically. Improve this skill instead of covering the content, encouraging analogical reasoning and clinical reasoning, increasing focus on the topic and the concept of learning, encouraging the students to apply the knowledge learned in practice, improving problem-solving skills and improving organizational performance (due to the need for Formulation of strategy, reorganization, and planning) help.

Overall, results of the study showed that the puzzle game-based training approach could be used in teaching sensitive and important surgeries such as CABG surgery for surgery and surgical technology students.

The strategy of the research team to reduce the effect of eight factors that threaten the internal validity of the quasi-experimental study is as follows:

### History

Lack of history of attending and working in the CABG operating room and not watching the CABG surgery video were among the inclusion criteria for our study. Also, the students in our study did not receive any training about CABG surgery except for the intervention of our study (puzzle game); Therefore, the history factor as a threatening factor for the internal validity of the quasi-experimental study has been controlled in our study.

### Maturation

Since the interval between the pre- and post-tests in our study was short (14 days) and the students in the study did not receive any training about CABG surgery except the intervention of our study (puzzle game); Therefore, the maturation factor as a threatening factor for the internal validity of the quasi-experimental study has been controlled in our study.

### Instrumentation

Since we used a type of test with similar questions to conduct pre- and post- tests; Therefore, the instrumentation factor as a threatening factor for the internal validity of the quasi-experimental study has been controlled in our study.

### Testing

Since we did not provide the students with the score of the test before the intervention and also the correct answers to the questions of this test, as well as the interval between the test before and after the intervention, in order to reduce the possibility of remembering the content of the questions of the test before the intervention (Recall Bias) was 14 days; Therefore, the testing factor as a threatening factor for the internal validity of the quasi-experimental study has been controlled in our study.

### Selection bias

Since we conducted a single-group study without a control group, there is no problem of differences between study groups in terms of study variables; But we have used a non-random sampling method (because our statistical population was limited and all students did not want to participate in the study, and according to the principles and rules of ethics in medical research, we were able to force students to attend We were not in the study); Therefore, the current research team has done as much as possible to control the selection bias factor as a risk factor of quasi-experimental study.

### Regression to the mean

This factor did not exist in our study, because we did not consider a specific score as a criterion in the analysis of the results of the students’ tests before and after the intervention, as well as the score of each student from the test after the intervention compared to the test before the intervention had increased.

### Social interaction

We had stated in the study contract with the students that after entering the study and until the end of the study, the student cannot read the CABG surgery training material and share it with others, and also the students cannot play the puzzle game and answer the questions get guidance and advice from others on the questions of the tests before and after the intervention and raise any problems in playing the game or answering the questions of the tests before and after the intervention only with the research team; Therefore, the social interaction factor as a risk factor for the validity of the quasi-experimental study has been controlled in our study.

### Attrition

18 students participated in the test before our intervention, and during the study period, 2 students who met one of the exclusion criteria (unwillingness to continue cooperation and play the puzzle game for 1 time), were withdrawn from our study, and 16 students participated in the post- test. In order to avoid the effect of the Austrian factor, the effect of the scores of the two students who were excluded from the study on the average scores of the pre-test was controlled using the covariance test so that it does not have a confounding effect on the results of our study; Therefore, the Austrian factor as a risk factor for the validity of the quasi-experimental study has been controlled in our study.

### Study implications

Based on the results of this study, the CABG surgery puzzle game can be used as a complementary tool for conventional training of CABG surgery principles. Since this game is online and can be used at any time and place and on Laptops, tablets and mobile phones are included and have the lowest cost. Therefore, a wide range of universities and students can use it to train surgical students and surgical technology as well as novice surgical technologists and establish educational justice to a large extent in the country. Also, this game can be used in situations where it is impossible to be present in the clinical environment, such as the Covid-19 pandemic. However, studies to evaluate the generalizability and the effect of using the CABG surgery puzzle game on the skills and clinical performance of students and comparing the puzzle game-based training method with the traditional training method in acquiring knowledge and skills, as well as the amount of consolidation of the learned material in the memory Learners are needed.

### Study strengths

Among the strengths of the present study are the use of the puzzle game-based training method and gamification in CABG surgery to train the scrub surgery technologists’ duties in each stage of the surgery mentioned above and the use of a particular standardized researcher-made test to measure knowledge and performance. The knowledge of surgical technology students about the clinical of scrub surgical technologists in each of the stages of CABG surgery, the use of a new, low-cost, online training method that can be used at any place and time and with any smart device (laptop, tablet and mobile phone) ) Cited.

### Study limitations

Among the study’s limitations, we can mention the lack of a control group, the small sample size due to limited access to students and the use of virtual exams due to the closure of universities due to the Covid-19 pandemic (despite the creation of virtual exam security).

## Conclusion

The results of the present study showed that the use of puzzle games in CABG surgery training led to a significant improvement in the knowledge and cognitive performance of surgical technology students regarding the stages of CABG surgery and its sequence, as well as the tools and equipment used in each stage of surgery and the sequence of their preparation.

It is possible to use puzzle game-based learning and gamification principles to prepare surgical technology students before entering the actual operating room and clinical environment were used as a supplementary training tool so that students could have a more effective presence in the CABG surgery room and learn supplementary material in that environment.

## Electronic supplementary material

Below is the link to the electronic supplementary material.

**Highlights**: Puzzle game-based learning: a new approach to promote learning of principles of coronary artery bypass graft surgery

## Data Availability

The datasets used during the current study available from the corresponding author on reasonable request.
