# Supplementary material for: Puzzle game-based learning: a new approach to promote learning of principles of coronary artery bypass graft surgery
Source: BMC Med Educ. 2023 Apr 13;23:241. doi: 10.1186/s12909-023-04156-w (PMC10100633; doi:10.1186/s12909-023-04156-w)
Supplement: Supplementary file 1 — Highlights: Puzzle game-based learning: a new approach to promote learning of principles of coronary artery bypass graft surgery [file 12909_2023_4156_MOESM1_ESM.docx]

**Highlights**

**Puzzle game-based learning: a new approach to promote learning of principles of coronary artery bypass graft surgery**

- The CABG surgery online puzzle game is designed to include different stages of surgery (from the stage of preparing the patient to the stage of sutures and dressing the surgical site and the tools used in each stage).
- The use of this puzzle game has led to the improvement of knowledge and cognitive function of operating room technology students.
- This puzzle game can be used as a complementary tool for training medical and operating room technology students to prepare them before entering the clinical environment.
